# Multireference Averaged Quadratic Coupled Cluster (MR-AQCC) Study of the Geometries and Energies for *ortho*-, *meta*- and *para*-Benzyne

**DOI:** 10.1021/acs.jpca.4c04099

**Published:** 2024-09-06

**Authors:** Khanh Vu, Joshua Pandian, Boyi Zhang, Christina Annas, Anna J. Parker, John S. Mancini, Evan B. Wang, Diomedes Saldana-Greco, Emily S. Nelson, Greg Springsted, Hans Lischka, Felix Plasser, Carol A. Parish

**Affiliations:** † Department of Chemistry, Gottwald Center for the Sciences, University of Richmond, Richmond, Virginia 23173, United States; ‡ Department of Chemistry and Biochemistry, Texas Tech University, Lubbock, Texas 79409, United States; § Department of Chemistry, Loughborough University, Ashby Road, Loughborough LE11 3TU, Leicestershire, U.K.

## Abstract

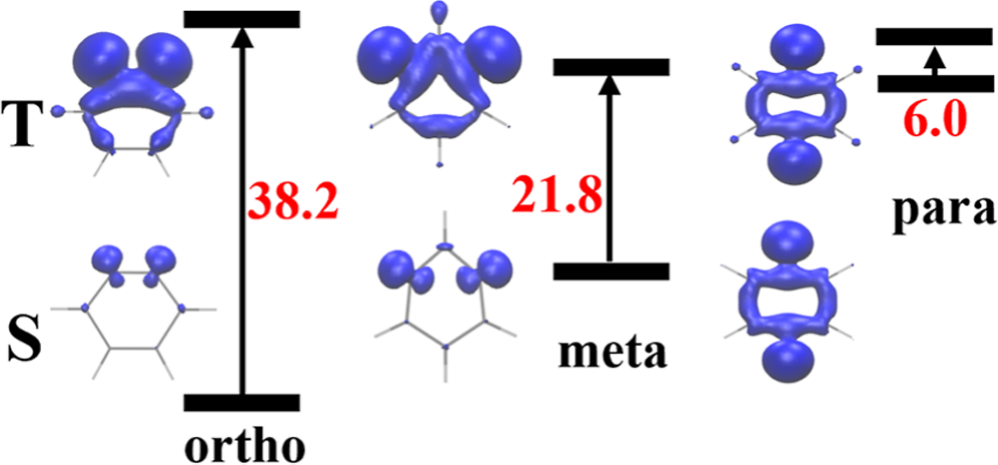

The diradical benzyne isomers are excellent prototypes for evaluating the ability of an electronic structure method to describe static and dynamic correlation. The benzyne isomers are also interesting molecules with which to study the fundamentals of through-space and through-bond diradical coupling that is important in so many electronic device applications. In the current study, we utilize the multireference methods MC-SCF, MR-CISD, MR-CISD+Q, and MR-AQCC with an (8,8) complete active space that includes the σ, σ*, π and π* orbitals, to characterize the electronic structure of *ortho-, meta-* and *para*-benzyne. We also determine the adiabatic and vertical singlet–triplet splittings for these isomers. MR-AQCC and MR-CISD+Q produced energy gaps in good agreement with previously obtained experimental values. Geometries, orbital energies and unpaired electron densities show significant through-space coupling in the *o*- and *m*-benzynes, while *p*-benzyne shows through-bond coupling, explaining the dramatically different singlet–triplet gaps between the three isomers.

## Introduction

**Figure 1:**
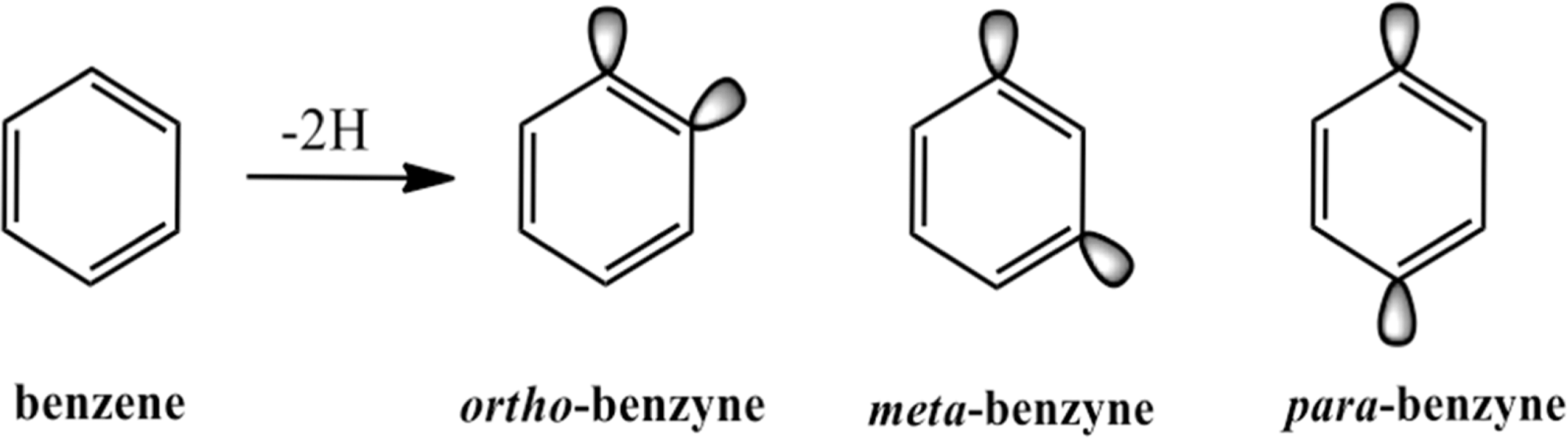
Didehydro-benzyne isomers of benzene.

The benzyne diradicals have been the subject of many experimental and computational studies due to their fundamental nature and unusual bonding, as well as their range of potential applications. For instance, the structure of the benzyne diradicals yields insight into the important behavior of polyaromatic hydrocarbons (PAHs) in combustion and soot.^1−7^ Naturally occurring antitumor molecules such as calicheamicin undergo the Bergman cyclization reaction to produce *para*-benzyne, motivating interest in these molecules for their potential use in cancer treatments.^8−38^ In addition, recent studies show the benzynes as building blocks for larger systems such as graphene.^39,40^ The three isomers of benzyne, *ortho* (*o*)-, *meta* (*m*)-, and *para* (*p*)-benzyne (Figure 1), are the subjects of this study.

Through-space and through-bond interactions between the radical electrons lead to a stabilized singlet state which falls below the triplet state in energy.^41,42^ The ability of the diradical to abstract hydrogen atoms from proximate sources such as DNA is believed to originate in the triplet state and therefore the singlet–triplet (S–T) energy gap is a measure of the hydrogen abstraction reactivity of the diradical.^43^ As a result, there have been many previous studies attempting to characterize the singlet–triplet splitting in all three isomers.^42,44−62^ The open-shell nature of these molecules makes for interesting electron interactions that are challenging to describe accurately. Indeed, the S–T gap and other characteristics of the benzynes are frequently used in benchmark studies for developing new computational methods.^44,52,54,63−72^ Early approaches using unrestricted Hartree–Fock methods proved to be insufficient in capturing the full extent of electron coupling interactions while density functional theory (DFT) and coupled-cluster methods were shown to be better.^25,29,44,45,55,59^ However, it is not clear if a density-based approach captures the physical nature of these diradicals, and therefore it cannot be known a priori whether DFT is an adequate method.^55,73^

**Figure 2:**
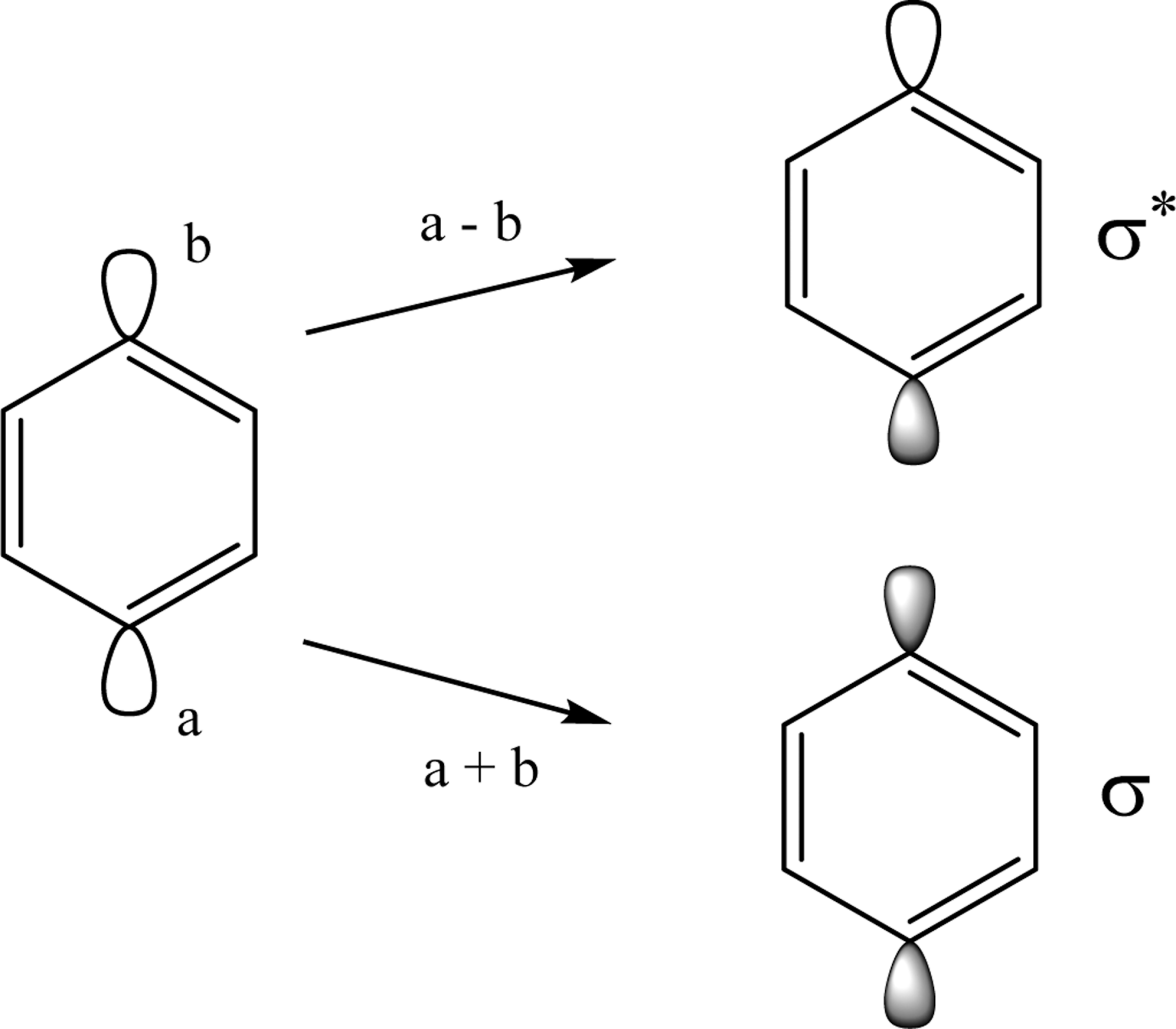
Formation of in-phase (σ) and out-of-phase (σ*) diradical lobes in *para* didehydro-benzyne.

For many diradicals, the difficulty in obtaining a proper theoretical characterization is due to the multiconfigurational nature of the molecules. If the diradical lobes are degenerate more than one electron configuration may be needed to fully capture the physical behavior of the system. For instance, in *p*-benzyne the dehydro lobes on C1 and C4 can combine to produce a σ and σ* orbital (Figure 2). If the σ and σ* orbitals are degenerate or nearly degenerate, then the configurations (σ)^2^, (σ*)^2^ and (σ)^1^ (σ*)^1^ need to be included in the reference wave function, i.e. this is a multiconfigurational molecule that needs a multireference wave function (MR) for proper characterization. In addition to the MR problem, polyradicals often have a high density of low lying electronic states, and spin contamination may occur where low lying states of different spin mix with the state being characterized.

Electronic structure methods have been developed to treat the multiconfigurational nature of diradicals. The Spin-Flip approach developed by Krylov and co-workers obtains multiconfigurational singlet states from single-configurational triplet states through the use of a spin operator, taking in both dynamical and nondynamical correlation effects.^54,74−87^ The Singlet-Type Strongly Orthogonal Geminal (SSG) method developed by Rassolov makes use of geminals^88,89^ in the atomic orbitals and takes correlation effects into account via Epstein-Nesbet perturbation theory.^90−93^ The Completely Renormalized Coupled Cluster (CR-CC(2,3)) method developed by Piecuch and Wloch renormalizes the traditional single-reference coupled-cluster method using a biorthogonal formulation.^94−96^ The state specific Mukherjee multireference coupled cluster singles and doubles (Mk-CCSD) method developed by Evangelista et al.^44,66,67^ is another example of a multireference coupled-cluster method. Density Cumulant Functional Theory (DCT) developed by Sokolov et al. has also been shown to take in account correlation effects and accurately describe diradical systems.^68,97−101^ Most recently, DFT approaches have been applied to the MR problem with the development of the state-averaged multiconfiguration pair-density functional theory,^102^ the renormalized singles plus the particle–particle Tamm-Dancoff approximation within the density functional approximation,^103^ and configurational interaction based on constrained DFT.^104^ As a general alternative to these methods traditional multireference (MR) configuration interaction (MR-CI) and the related MR-AQCC method^105,106^ allow flexible choices of wave functions and application to virtually any desired spin multiplicity.^107^ A major advantage of MR-CI and MR-AQCC is their variational nature in which the methodological treatment can be systematically improved, and analytic energy gradients are easily accessible.

An important consequence of the different radical locations in the three benzyne isomers is that they have different energy gaps between the lowest lying singlet state and triplet state. Wenthold et al.^56^ showed via photoelectron spectroscopy that *o-*benzyne has the largest S–T gap (37.5 kcal/mol). Radical electron coupling was attributed mainly to through-space interactions due to the proximity of the radicals.^8,41,42^ In comparison, *p-*benzyne has the smallest S–T gap (3.8 kcal/mol) attributed mainly to through-bond interactions.^8,41,42,56^ Clark and Davidson^108^ analyzed *p*-benzyne derivatives via a CASSCF/cc-pVDZ treatment, and identified many with exceptionally small singlet–triplet splitting energies, and even a few with a triplet ground state. *m-*benzyne, with a S–T gap of 21.0 kcal/mol, falls in the middle of the other two isomers.^56^ A valence bond study performed by Wei et al. in 2009 suggested that stabilization of the singlet state in *m-*benzyne due to through-bond interactions is slightly (10%) more than through-space interactions.^109^

**Figure 3:**
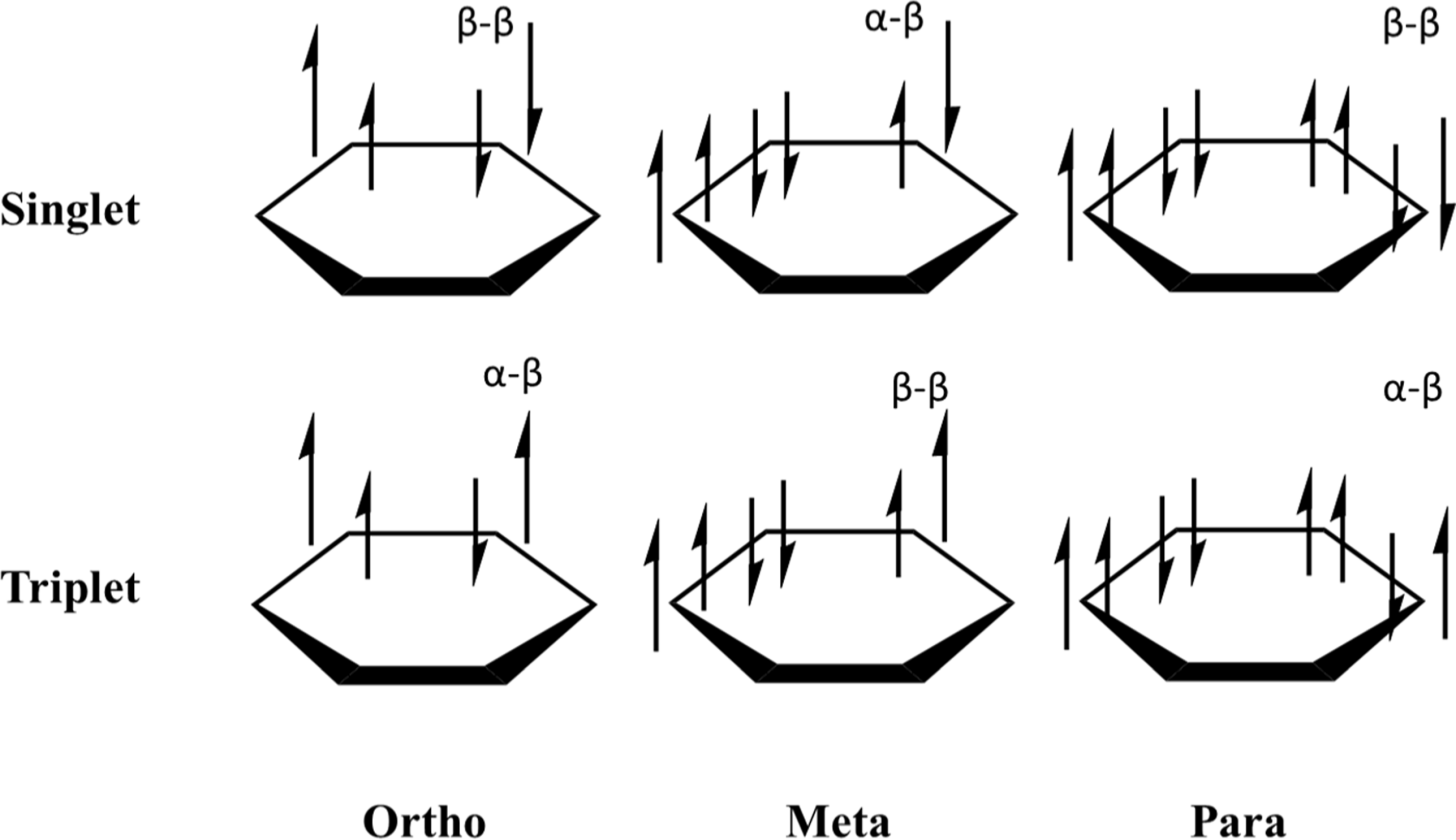
Spin polarization for the singlet and triplet states of the benzyne isomers. Electrons are represented according to the intraatomic Hund’s rule and the pair coupling principle.

Unpaired electrons can interact via spin polarization. In addition to through-space and through-bond interactions, Crawford et al.^110^ have used this phenomenon to explain the singlet stabilization in *p-*benzyne. A representation of the spin polarization for the singlet and triplet states of all three benzyne isomers is shown in Figure 3. Spin polarization effects are governed by the intraatomic Hund’s rule as well as the electron coupling in bond pairs.^111^ These rules dictate that electrons in orthogonal orbitals on the same atom be spin aligned (α–α or β–β interactions) while bonding electrons shared between atoms must be spin paired (α–β interactions). In *o-* and *p-*benzyne, there are two stabilizing β–β interactions on the radical centers in the singlet state while there is one β–β interaction and one destabilizing α–β interaction in the triplet state. In *m-*benzyne, however, there are two β–β interactions in the triplet state rather than the singlet state. Thus, spin polarization plays a role in singlet stabilization for *o-* and *p-*benzyne but not *m-*benzyne.

Benzyne isomer geometries in the singlet state have been studied using a myriad of approaches, including SCF, DFT, and CCSD methods.^44−51,53,54,61,108,112^ Fewer studies have been done on the triplet state, and include an *o-*benzyne SCF geometry optimization by Scheiner et al.,^53^ a study by Slipchenko and Krylov using a Spin-Flip-DFT approach on all three isomers,^54^ a study by Debbert and Cramer using various theoretical approaches,^30,61^ and an analysis of the three isomers by Clark and Davidson using the CASSCF method.^57^ A topic of special interest is the accurate determination of the singlet state structure of *m-*benzyne and the geometry implications of the radical carbon interactions. Kraka et al.^47^ showed that restricted density functional methods tended to characterize singlet *m-*benzyne as a bicyclic structure with a bond between the C1 and C3 radical carbons while unrestricted DFT methods as well as coupled-cluster methods show a monocyclic structure. Winker and Sander^113^ also performed geometry optimizations at various levels of theory and confirmed the monocyclic structure of singlet *m-*benzyne. Thus, like S–T gaps, the C1–C3 distance has become an indicator of how well a method captures the multiconfigurational nature of this particular diradical.

Our work focuses on characterizing the energetics and geometries of the singlet and triplet states of *o-*, *m-*, and *p-*benzyne using a highly correlated, multireference approach. Multiconfigurational self-consistent field (MCSCF) is a method that can be used for molecules with low-lying ground and excited states. To overcome the static correlation problem brought on by the open-shelled nature of diradicals, various multireference methods, as already introduced above, can be employed. The multireference configuration interaction with singles and doubles (MR-CISD) method^114^ can be used along with the state specific multireference average quadratic coupled cluster (MR-AQCC) method.^105,106,115^ The MR-AQCC method is of special interest since it allows a balanced description of quasi-degenerate configurations in the reference wave function and of dynamic electron correlation including size-extensivity corrections. These methods were used in a previous study by Wang et al.^55^ to study the vertical gaps and other properties of *p-*benzyne and proved to properly capture the physical nature of the benzyne diradicals. Recently, MR-AQCC has also been used for the calculation of acenes and periacenes.^39,116^ The biradical character of zethrenes,^117^ pyrazine^118^ and anthracene^119^ have been studied in this way. Also, the antiferromagnetic coupling in non-Kekulé dimethylenepolycyclobutadienes, taking into account spin multiplicities up to septets, has been investigated.^120^

Hanauer and Köhn^60^ performed an analysis of singlet–triplet splitting energies of the benzynes using MR-CCSD(T) with correlation-consistent double and triple-ζ basis sets (cc-pVDZ/TZ). However, Wang et al. demonstrated that the CAS(2,2) active space used by Hanauer and Köhn was inadequate for the characterization of *p*-benzyne due to interactions with the π orbitals. Wei et al.^109^ in 2009 demonstrated that the inadequacy of the CAS(2,2) active space for *m*-benzyne is mostly due to through-bond interactions. In this work, we use a CAS(8,8) active space that includes all of the π orbitals for all the benzyne isomers in order to capture these effects.

To the best of our knowledge, this is the first MR-AQCC analysis of the geometries and energies of all of the benzyne isomers. MR-AQCC is a genuine multireference method allowing flexible incorporation of quasi-degenerate configurations into the reference space to account for static electron correlation. It stands out from other quantum methods as it not only includes dynamic electron correlation using single and double excitations into the virtual space, but also includes coherent size-consistency contributions and is free of spin contamination.

## Methods

**Figure 4:**
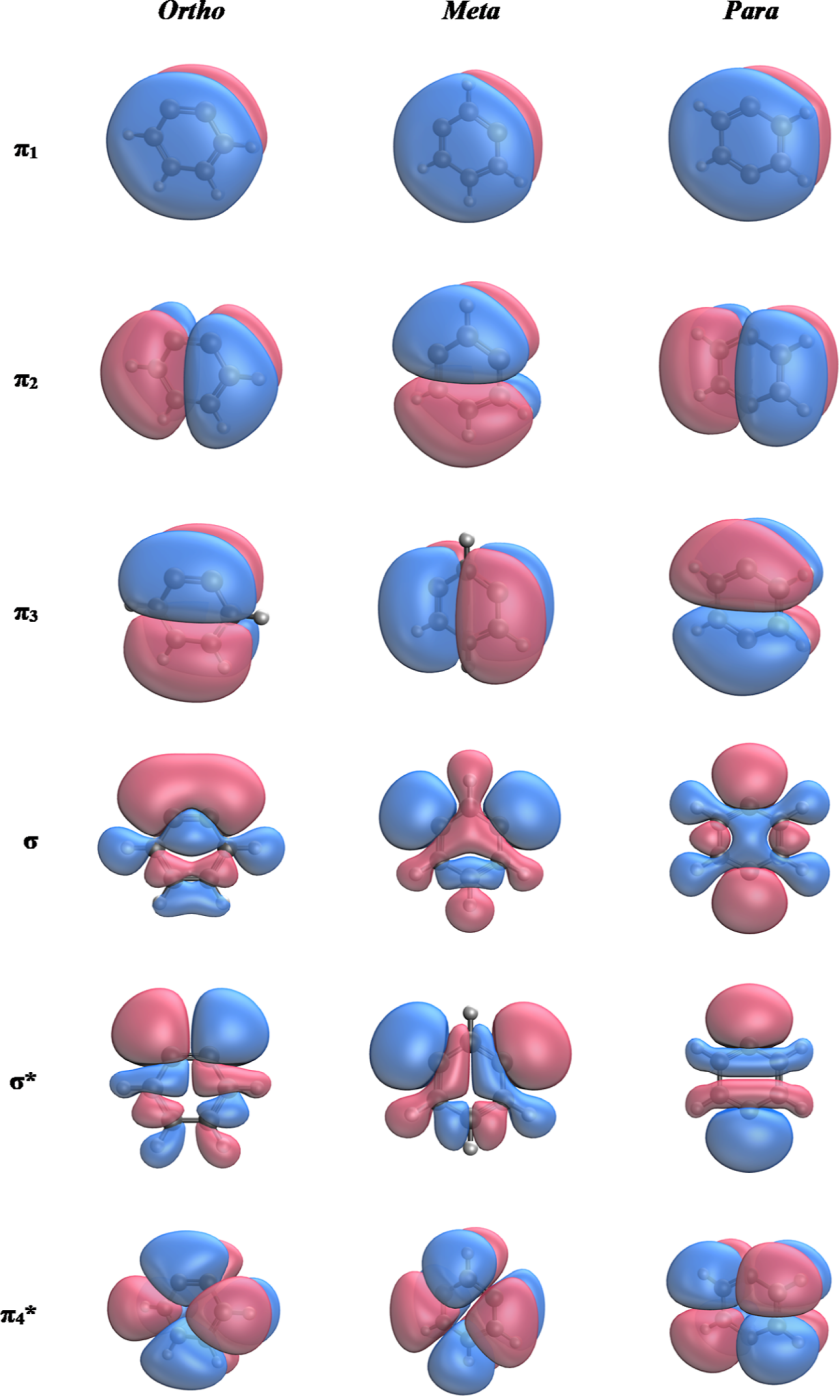
Active space molecular orbitals for the benzyne isomers optimized at the MCSCF/cc-pVTZ level of theory.

Initial geometry optimizations on the triplet states of *o*-, *m*-, and *p-*benzyne were performed using the UB3LYP method with a 6-31G** basis set in Q-Chem.^121^ A single point calculation was then performed using the restricted open-shell Hartree–Fock (ROHF) method with the cc-pVDZ basis set. The molecular orbitals from these calculations were characterized based on *D*_2*h*_ (*p*-benzyne) and *C*_2*v*_ (*o*- and *m*-benzyne) symmetry and used to determine the orbital space for subsequent calculations. Orbitals were identified as either doubly occupied (DOCC) or part of the complete active space (CAS). The CAS was used to construct all possible configuration state functions for any particular state symmetry. MCSCF, MR-CI and MR-AQCC geometry optimizations were performed with a CAS(8,8) active space. This included two radical electrons occupying the σ and σ* orbitals on the didehydrocarbons and six electrons occupying the pi and pi* orbitals. The symmetries of these orbitals are characterized and shown in Table 1. Figure 4 shows the eight CAS orbitals for the three benzyne isomers.

**Table 1:** CAS Molecular Orbital Symmetries for the Benzyne Isomers

*o*-benzyne (*C*_2*v*_)	1b_1_(π_1_)	1a_2_(π_2_)	2b_1_(π_3_)	10a_1_(σ)	8b_2_(σ*)	3b_1_ (π_4_*)	2a_2_(π_5_*)	3a_2_(π_6_*)
*m*-benzyne (*C*_2*v*_)	1b_1_(π_1_)	2b_1_(π_2_)	1a_2_(π_3_)	11a_1_(σ)	7b_2_(σ*)	3b_1_(π_4_*)	2a_2_(π_5_*)	5b_1_(π_6_*)
*p*-benzyne (*D*_2*h*_)	1b_3u_(π_1_)	1b_1g_(π_2_)	1b_2g_(π_3_)	6a_1g_(σ)	5b_1u_ (σ*)	1a_u_(π_4_*)	3b_3u_(π_5_*)	2b_2g_(π_6_*)

The lowest lying singlet and triplet states were calculated independently for each isomer. In *o-* and *m-*benzyne, the lowest lying singlet states have ^1^A_1_ symmetry, while the lowest lying triplet states have ^3^B_2_ symmetry. For *p-*benzyne, the lowest lying singlet state has ^1^A_g_ symmetry, and the lowest lying triplet state has ^3^B_3u_ symmetry.

The MCSCF wave functions were used to provide the MOs for the MR-CISD and MR-AQCC calculations. Electrons in six core orbitals (1–3a_1_ and 1–3b_2_ for *o-*benzyne, 1–4a_1_ and 1–2b_2_ for *m-*benzyne, 1–2a_g_, 1–2b_1u_, 1b_2u_, 1b_3g_ for *p-*benzyne) were frozen. The problem of size-extensivity associated with configuration interaction calculations was addressed a posteriori with the Davidson correction using the Pople method (MR-CISD+Q).^122^

Although there have been analyses on the complete active orbital set of *p-*benzyne,^108,110^ to the best of our knowledge, the orbitals for *o-* and *m-*benzyne have not been previously reported. The MCSCF orbital energies for the CAS will be discussed in the Results section.

Both *o-* and *m-*benzyne were optimized with *C*_2*v*_ geometry with the yz plane serving as the molecular plane and the *z*-axis serving as the C_2_ rotation axis. *para*-benzyne was optimized with *D*_2*h*_ geometry in the yz molecular plane. All methods were used in combination with the correlation consistent cc-pVDZ and cc-pVTZ basis sets developed by Dunning.^123,124^ The geometries optimized from the MR-AQCC/cc-pVTZ calculation were used to perform single point energy calculations at the MCSCF, MR-CISD, and MR-AQCC levels to obtain vertical S–T excitation energies. The calculations were performed using the COLUMBUS 7.0 program^125−128^ with the DALTON atomic orbital integral package.^129^

The effective unpaired electron densities (UED)^130,131^ and Mulliken populations were determined for the MR-AQCC/cc-pVTZ singlet and triplet state geometries. A nonlinear model as suggested by Head-Gordon^132^ was used to obtain the UED so that the contribution from the nearly occupied and unoccupied natural orbitals is reduced.^39,131^

## Results and Discussion

### Energy Gaps and Electron Configurations

**Figure 5:**
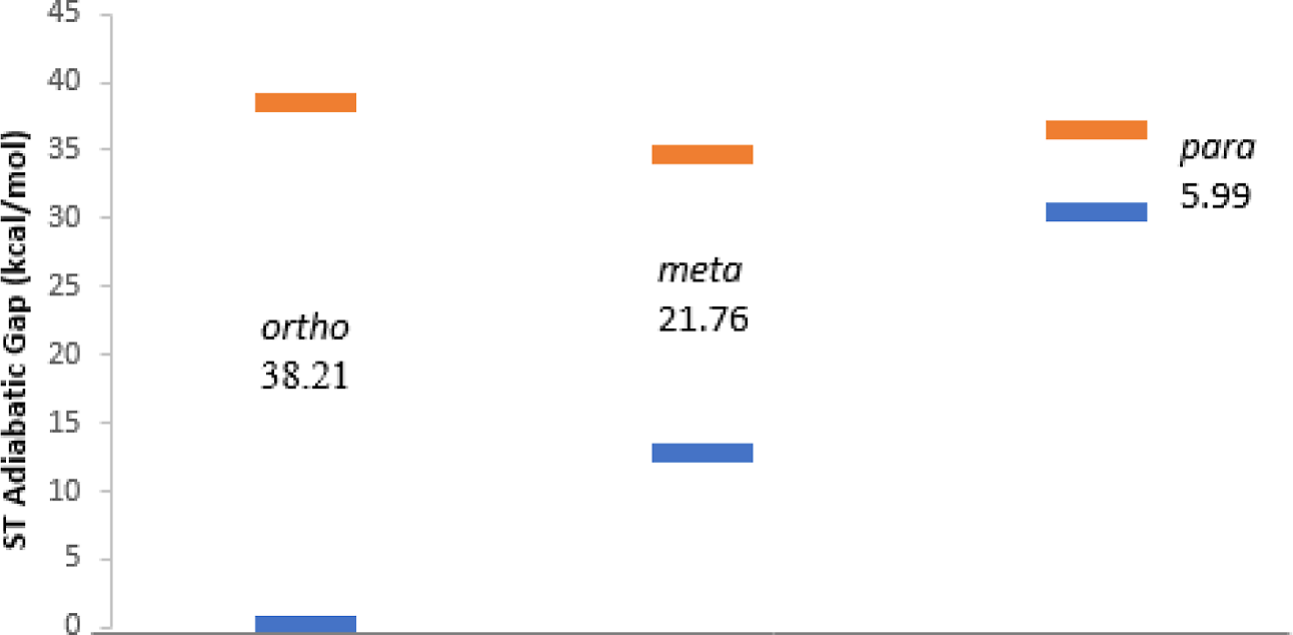
Relative energies of the benzyne isomers from AQCC/cc-pVTZ geometry optimizations. Singlet ground state relative energies are in shown in blue while triplet state relative energies are in orange.

The adiabatic S–T gaps at each level of theory, along with the dominant electron configurations from the MR-AQCC calculations for the singlet and triplet state of each isomer, are shown in Tables 2–4. For the singlet states, we find that the weight (c^2^) of the π_1_^2^ π_2_^2^ π_3_^2^ σ^2^ (σ* for para) configuration is 64.8, 62.3 and 48.5% in *o-*, *m-* and *p-*benzyne, respectively. The second configuration, π_1_^2^ π_2_^2^ π_3_^2^ (σ*)^2^ (σ for para), occurs with significantly reduced (*c*^2^) values of 4.1, 6.7 and 19.5% for *o-*, *m-* and *p-*benzyne, with *p*-benzyne showing the strongest open-shell character. Analysis of the remaining occupations beyond this reference space contain configuration coefficients that when squared contribute less than one percent. This confirms that the CAS(8,8) reference space used in this study is capturing well the open shell character. This provides a balanced basis for calculating the total electron correlation energy involving single and double excitations into the virtual orbital space derived from the CAS(8,8) reference configurations. As expected, all of the isomeric triplets are single configurational(π_1_^2^ π_2_^2^ π_3_^2^ σ^1^ (σ*)^1^). The weighting of the second configuration in the singlet state correlates with (a) the decrease in stability of the singlet (vide infra; Figure 5), (b) a decrease in the S–T gap (vide infra; Figure 5) and (c) a narrowing of the σ and σ* orbital energy gap (vide infra; Table 6) as the radical electrons move further apart in the *o*-, *m*- and *p*-benzyne isomers. The multiconfigurational wave function that results serves as affirmation for our multireference approach to the problem.

**Table 2:** *ortho*-Benzyne MCSCF, MR-CISD, MR-CISD+Q, and MR-AQCC Adiabatic (Top) and Vertical (Bottom) Gaps (kcal/mol) From the Singlet A_1_ Ground State Using a CAS(8,8) Reference Wave Function and the cc-pVTZ Basis Set

	dominant configuration	MCSCF	MR-CISD	MR-CISD+Q	AQCC	exp^a^
**state**	in AQCC	*E*_exc_	*E*_exc_	*E*_exc_	*E*_exc_	*E*_exc_
^**1**^**A**_**1**_^b^	64.8% π_1_^2^ π_2_^2^ π_3_^2^ σ^2^					
	4.1% π_1_^2^π_2_^2^π_3_^2^(σ*)^2^					
^**3**^**B**_**2**_	68.4% π_1_^2^π_2_^2^π_3_^2^σ^1^(σ*)^1^	35.50	38.08	38.34	38.21	37.5
	Vertical Gaps	51.22	53.09	53.54	53.81	N/A

a Wenthold, Squires, Lineberger. J. Am. Chem. Soc. 120, 5279 (1998).

b All single point absolute energies included in the Supporting Information Total ground state geometry optimization energies (au): −229.5977951 (MCSCF), −230.3056689 (MR-CISD), −230.4565400 (MR-CISD+Q), and −230.4496720 (MR-AQCC).

**Table 3:** *meta*-Benzyne MCSCF, MR-CISD, MR-CISD+Q, and MR-AQCC Adiabatic (Top) and Vertical (Bottom) Gaps (kcal/mol) from the Singlet A_1_ Ground State Using a CAS(8,8) Reference Wavefunction and the cc-pVTZ Basis Set

	dominant configuration	MCSCF	MR-CISD	MR-CISD+Q	AQCC	exp^a^
state	in AQCC	*E*_exc_	*E*_exc_	*E*_exc_	*E*_exc_	*E*_exc_
^1^A_1_^b^	62.3% π_1_^2^π_2_^2^π_3_^2^σ^2^					
	6.7% π_1_^2^π_2_^2^π_3_^2^(σ*)^2^					
^3^B_2_	68.7% π_1_^2^π_2_^2^π_3_^2^σ^1^(σ*)^1^	15.75	19.93	21.92	21.76	21.0
	Vertical gaps	31.28	33.39	34.92	35.02	N/A

a Wenthold, Squires, Lineberger. J. Am. Chem. Soc. 120, 5279 (1998).

b All single point absolute energies included in the Supporting Information. Total ground state geometry optimization energies (au): −229.5703556 (MCSCF), --230.2822778 (MR-CISD), −230.4366920 (MR-CISD + Q), −230.4295250 (MR-AQCC).

**Table 4:** para-Benzyne MCSCF, MR-CISD, MR-CISD + Q, and MR-AQCC Adiabatic (Top) and Vertical (Bottom) Gaps (kcal/mol) from the Singlet A_1_ Ground State Using a CAS(8,8) Reference Wave Function and the cc-pVTZ Basis Set^a^

state	dominant configuration	MCSCF	MR-CISD	MR-CISD + Q	AQCC	exp^b^
	in AQCC	*E*_exc_	*E*_exc_	*E*_exc_	*E*_exc_	*E*_exc_
^1^A_g_^b^	48.5% π_1_^2^ π_2_^2^ π_3_^2^ (σ*)^2^					
	19.5%π_1_^2^π_2_^2^π_3_^2^(σ)^2^					
^3^B_1u_	69.5% π_1_^2^ π_2_^2^ π_3_^2^ σ^1^ (σ*)^1^	2.66	3.77	5.10	5.99	3.8
	Vertical Gaps	4.72	5.76	6.49	7.61	N/A

a Wenthold, Squires, Lineberger. J. Am. Chem. Soc. 120, 5279 (1998).

b All single point absolute energies included in the Supporting Information. Total ground state geometry optimization energies (au): −229.5510824 (MCSCF), −230.2566518 (MRCISD), −230.4076352 (MRCISD+Q), and −230.4014864 (MR-AQCC).

The S–T gap is an indication of the extent of radical interaction, and having a singlet lie lower in energy than a triplet attests to singlet state stabilization. *o-*benzyne, having radical centers that are closest to each other, has the largest gap, where in contrast *p-*benzyne, having radical centers that are farthest apart, has the smallest gap. Figure 5 shows the relative energies of the benzyne isomers. It is evident that the singlet states are destabilized as the distance between radical centers increase from *o-* to *p*-benzyne. Given that the experimental heats of formation are known for the benzyne isomers, we can use these values (107.3 ± 3.5, 121.9 ± 3.1, and 138.0 ± 1.0 kcal/mol for *ortho*-, *meta*-, and *para*-benzyne, respectively)^36,37,56,133,134^ to see that our AQCC/cc-pVTZ relative energies for the singlets are quite accurate (Figure 5). For instance, using our AQCC/TZ geometry optimized singlets, we calculate the difference in energy for ortho–meta, meta–para and ortho-para as 12.6, 17.6, and 30.2 kcal/mol. These can be compared to the relative experimental energy differences of 14.6, 16.1, and 30.7 kcal/mol. We can also combine the experimental absolute heats of formation with the calculated singlet–triplet gaps to arrive at the energies of the isomeric triplets (Figure S1). This approach suggests that the triplet isomers are all relatively close in energy, i.e. 141.8, 142.9, and 144.8 kcal/mol for para, meta and *ortho*-benzyne. This is perhaps not surprising that the triplet relative energies would be more isoenergetic than the singlet energies where through-space and through-bond coupling play a more discriminating role in the stability of the various isomers.

As the level of theory increases, and dynamical electron configuration is included, the calculated adiabatic gap approaches the experimental value. The good agreement between our CISD + Q and AQCC results suggests an accurate size-extensivity correction. In *p-*benzyne, the MR-AQCC overestimates the S–T gap.

The AQCC/TZ singlet state geometries were used to compute vertical S–T gaps (Tables 2–4). The gaps are higher than the adiabatic gaps, which is to be expected. The magnitude of the change reflects the importance of geometry relaxation and the need to use a computational method that captures the multiconfigurational nature of the isomers. The difference between the vertical and adiabatic gap for *p-*benzyne (Table 4) is significantly smaller than for *o-* and *m-*benzyne (Tables 2 and 3, respectively). This is due, in part, to the similar geometry for the singlet and triplet state of *p-*benzyne whereas in *o-* and *m*-benzynes the geometries differ significantly (vide infra).

Table 5 compares our Δ*E*(ST) results to splittings obtained with other methods as reported in the literature. Judging by the wave functions shown in Tables 2–4, *o-*benzyne displays the least diradical character of any of the benzynes, and therefore previous methods that had difficulty characterizing the *p-* and *m-* benzynes generally perform well for the *o*-benzyne isomer. Spin-flip-optimized orbital coupled-cluster doubles (SF-OD) is the most accurate in this case, with the MR-CI and MR-AQCC methods also showing good agreement with experiment, especially for *o*- and *m*-benzyne.

**Table 5:** Comparison of Computed and Measured ST Splittings for the Benzyne Isomers^a^

	ortho	meta	para
**MR-CISD/cc-pVTZ**	**38.1**	**19.9**	**3.8**
**MR-CISD+Q/cc-pVTZ**	**38.3**	**21.9**	**5.1**
**MR-AQCC/cc-pVTZ**	**38.2**	**21.8**	**6.0**
MkCCSD/cc-pVDZ^65^	35.1	18.7	4.5
CCSD(T)/pVTZ^58^	35.3	20.7	2.3
REKS/6-31G(d)^135^	36.5	21.6	4.1
SF-OD/cc-pVTZ^54^	37.6	19.3	3.9
SF-BHLYP^59^	37.3	18.8	4.0
SGHF/cc-pVTZ^52^	33.4	14.0	6.8
exp^56^	37.5	21.0	3.8

a Results From the Current Study Shown in Bold.

### Orbital Energies

The molecular orbitals were optimized at the MC-SCF(8,8)/cc-pVTZ level of theory. These orbital energies (Table 6) provide further explanation for the variation in isomeric singlet state energies and subsequent singlet–triplet gaps (Tables 2–4). A visualization of these orbitals can be seen in Figure 4. As the radical electrons move further apart in space, the two-configurational nature of the isomer increases, i.e. the ratio of the configuration weights π_1_^2^ π_2_^2^ π_3_^2^ σ^2^: π_1_^2^ π_2_^2^ π_3_^2^ (σ*)^2^ is 65:4 for *o*-benzyne; 62:7 for *m*-benzyne and 49:20 for *p*-benzyne. As expected, in the singlet state of both *o-* and *m-*benzyne, the π and σ orbitals are occupied and thus have a negative energy, while the π* and σ* orbitals are positive reflecting the decreased probability of being occupied. In the triplet state of *o-* and *m-*benzyne, there is electron occupation in the π, σ and σ* orbitals. In the *p-*benzyne singlet state, the antisymmetric σ* radical orbital falls lower than the symmetric σ orbital. This is indicative of through bond coupling as described by Hoffmann et al.^42^ and Crawford et al.^110^ Through bond coupling results from the mixing of unpaired/nonbonded σ electrons with the σ paired electrons in the ring. In *para*-benzyne, the radical electrons are separated by three intervening σ bonds and the mixing symmetry of this arrangement stabilizes the antisymmetric σ* orbital. This causes the σ* orbital to lie lower than the σ orbital in *para*-benzyne but not the other isomers.^41,42^ In the *p-*benzyne singlet, the energies for the σ and σ* orbitals are both negative reflecting the increased two-configurational nature of this isomer. This leads to a destabilization of this singlet isomer and subsequent narrowing of the singlet–triplet gap (Figure 5).

**Table 6:** Orbital Energies (au) of the Lowest Lying Singlet and Triplet for the benzynes Calculated at the MCSCF Level with a cc-pVTZ Basis Set

***o****-*benzyne	1b_1_(π_1_)	1a_2_(π_2_)	2b_1_(π_3_)	10a_1_(σ)	8b_2_(σ*)	3b_1_(π_4_*)	2a_2_(π_5_*)	3a_2_(π_6_*)
singlet	–1.020	–0.670	–0.677	–0.753	0.201	0.294	0.394	0.885
triplet	–1.005	–0.671	–0.657	–0.401	–0.214	0.316	0.319	0.834
***m*-benzyne**	**1b**_**1**_**(π**_**1**_**)**	**2b**_**1**_**(π**_**2**_**)**	**1a**_**2**_**(π**_**3**_**)**	**11a**_**1**_**(σ)**	**7b**_**2**_(σ*)	**3b**_**1**_**(π**_4_*)	**2a**_**2**_**(π**_5_*)	**5b**_**1**_**(π**_6_*)
singlet	–1.022	–0.687	–0.658	–0.528	0.046	0.307	0.356	0.840
triplet	–1.006	–0.668	–0.658	–0.372	–0.218	0.314	0.328	0.848
***p*-benzyne**	**1b**_**3u**_**(π**_**1**_**)**	**1b**_**1g**_**(π**_**2**_**)**	**1b**_**2g**_**(π**_**3**_**)**	**6a**_**1g**_**(σ)**	**5b**_**1u**_(σ*)	**1a**_**u**_**(π**_4_*)	**3b**_**3u**_**(π**_5_*)	**2b**_**2g**_**(π**_6_*)
singlet	–1.006	–0.669	–0.656	–0.210	–0.370	0.300	0.341	0.843
triplet	–1.007	–0.673	–0.655	–0.262	–0.313	0.303	0.342	0.840

An analysis of the NO populations of the isomeric MR-AQCC/TZ singlets confirms that σ orbitals not corresponding to the radical electrons are all either close to doubly occupied or unoccupied (with an NO population below a threshold of 0.03e). This confirms that σ orbitals other than those in Figure 2 do not contribute to the open shell character of these diradical isomers. The electron correlation of these orbitals and their through-bond interactions are thus well described by the single and double excitations into the virtual orbital space.

### Geometries

The AQCC/TZ geometry optimized structures are shown in Figure 7. Several differences in the singlet and triplet geometries provide evidence for the radical electron interaction present in the benzyne isomers. In general, the geometry of the triplet states are much closer to that of benzene.^50,55^ For *o*-benzyne, the shortening of the C1–C2 bond in the singlet state geometry is apparent: at the MR-AQCC/cc-pVTZ level, the C1–C2 bond is 1.26 Å while in the triplet state the C1–C2 bond is 1.40. The singlet state is much more distorted relative to benzene, and less symmetrical, due to the coupling between the didehydrocarbon atoms. The shortening of the C1–C2 bond in the singlet state geometry results in a distortion of the bond angles, with an increase in the C1–C2–C3 angle (involved in bond shortening) and a decrease in the C2–C3–C4 angle. The nature of the C1–C2 bond in *o-*benzyne has been discussed in previous studies.^46,50,51,53,57^ These prior works all report the C1–C2 bond to be between 1.25 and 1.26 Å, which is in agreement with our results. However, different interpretations are given as to whether the length of this bond constitutes a full triple bond.

The singlet and triplet geometries of *m-*benzyne (Figure 7) show a similar trend whereby the singlet is significantly more distorted and less symmetrical than the triplet. In the singlet state geometry, the through-space interaction between the didehydro carbon atoms pinches together the C1–C3 distance, even though these atoms are not directly bonded together. This effect of radical electron interaction can be seen in the angle distortion in the C1–C2–C3 angle. Compared to the angle of 115.2° degrees for the triplet state, the singlet state has an angle of 99.0°. The monocyclic/bicyclic structure of *m-*benzyne has become a standard benchmark test for the ability of a computational quantum method to properly describe a multiconfigurational molecular system. Higher level multireference approaches describe *m-*benzyne as a monocyclic structure rather than a bicyclic structure containing a C1–C3 bond.^44,47^Table 7 shows a comparison of our C1–C3 bond length results at the cc-pVTZ level with previously published distances. It is apparent that the multireference approach taken in this study accurately characterizes the structure of *m-*benzyne.

**Table 7:** Comparison of Didehydrocarbon (C1–C3) Distances (Å) for Singlet States of *meta*-Benzyne^a^

method	C1–C3 distances	ref
RHF-CCSD/cc-pVTZ	1.564	(44)
Mk-MRPT2/cc-pVTZ	1.873	(44)
Mk-MRCCSD/TCSCF	2.014	(67)
REKS(2,2)/6-31G**	2.051	(47)
**MR-AQCC/cc-pVTZ**	**2.088**	
**MR-CISD/cc-pVTZ**	**2.097**	
ODC-12/cc-pCVTZ	2.101	(68)
UB3LYP/6-31G**	2.136	(47)
**MCSCF/cc-pVTZ**	**2.174**	
CASSCF(8,8)/6-31G*	2.198	(47)

a Distances calculated as part of this study are shown in bold.

Important distances and angles of *p-*benzyne are also presented in Figure 7. The difference between the singlet and triplet geometries of *p-*benzyne is much smaller when compared to the same differences for *o-* and *m-*benzyne. There is no significant change in the bond distances, and the angles of the radical carbons only change by a few degrees.

**Figure 6:**
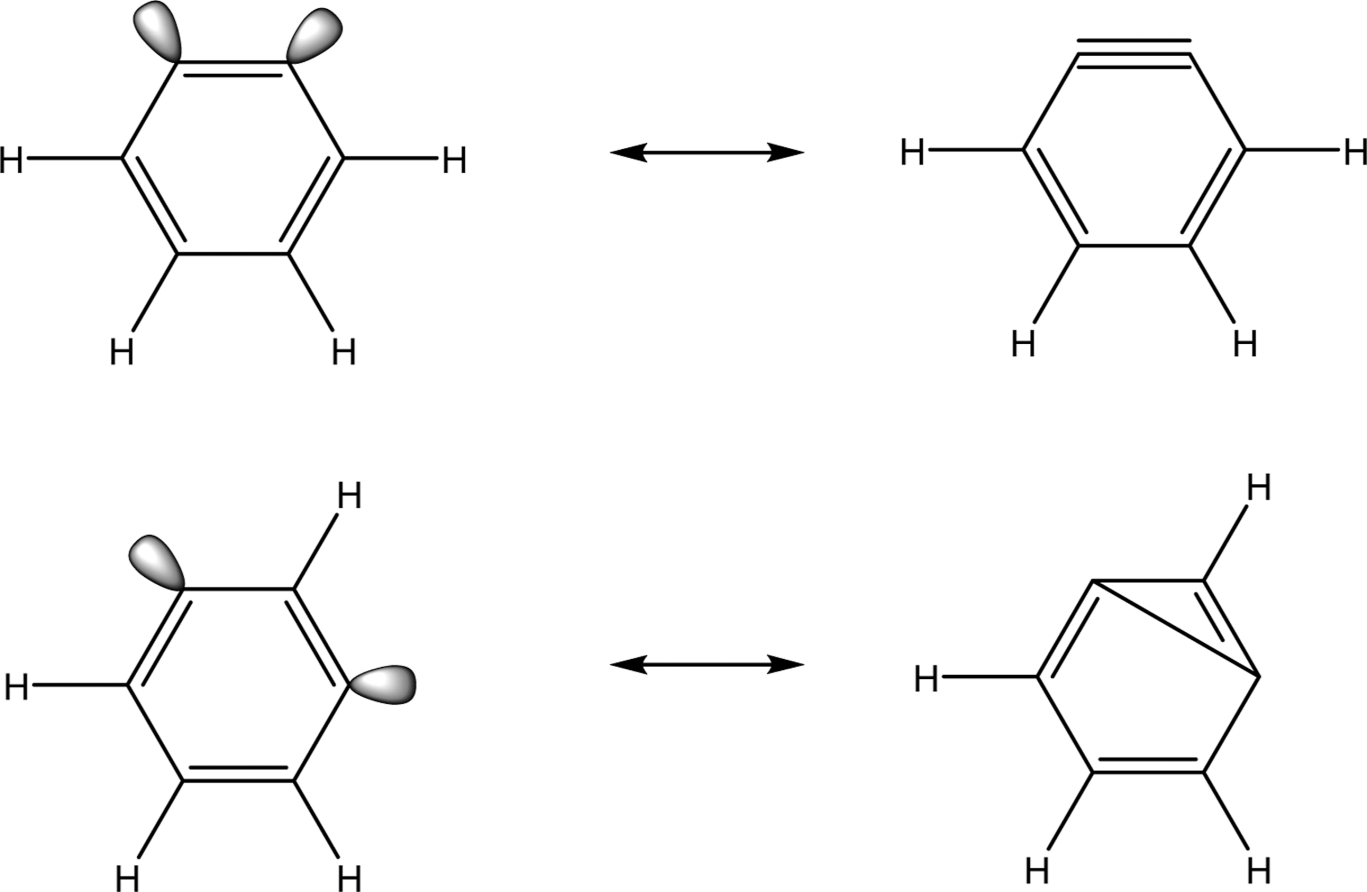
Resonance structures for *o*- and *m*-benzyne showing through-space coupling that leads to these diradicals having a fairly closed-shell nature.

**Figure 7:**
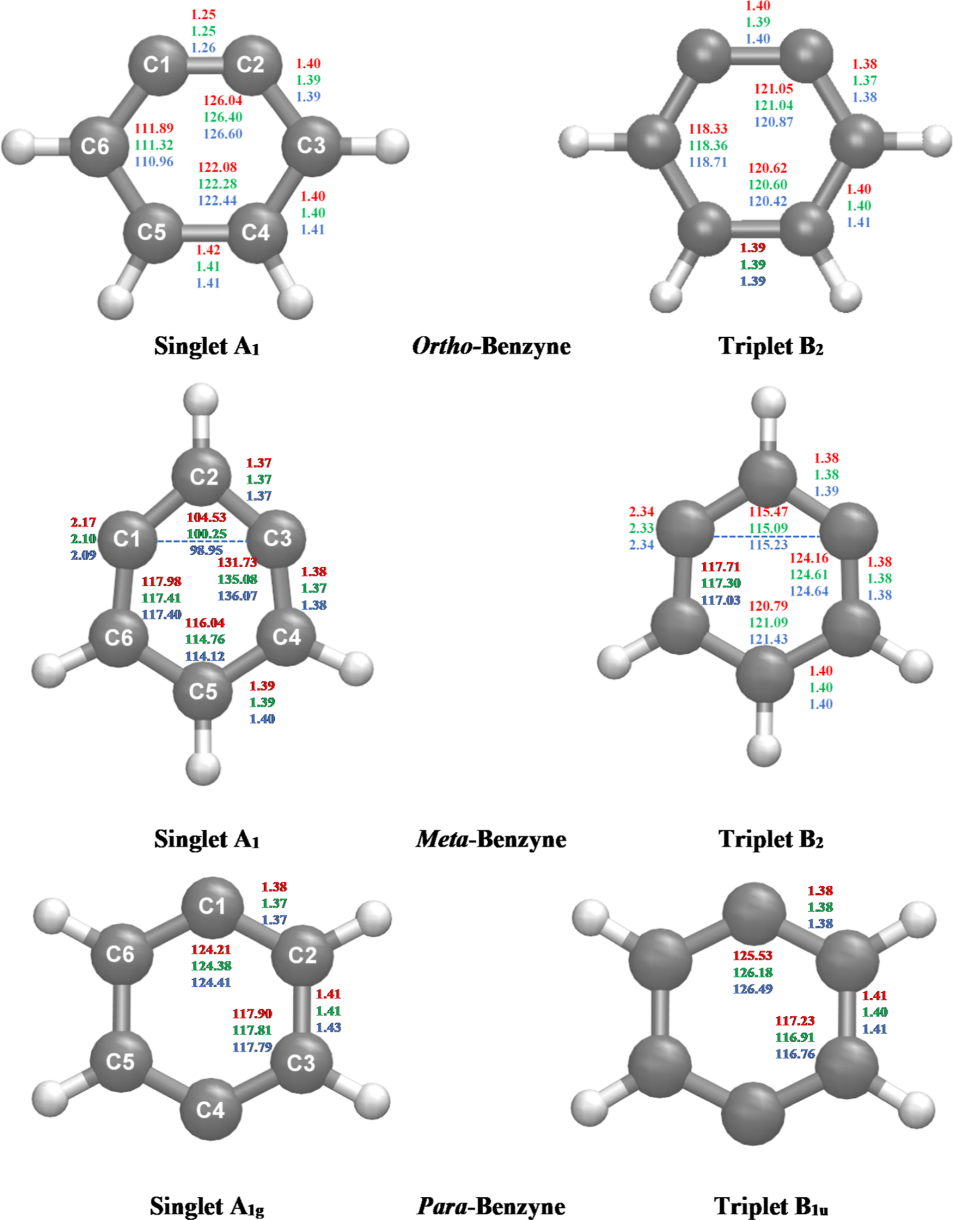
Geometry optimized structures of the benzyne isomers. Distances (Å) and angles (deg) are shown at the MCSCF (red), MRCI (green), and MR-AQCC (blue) levels of theory with the cc-pVTZ basis set.

The MC-SCF optimized MOs (Figure 4) and AQCC geometries (Figure 7) provide insight into the underlying differences between the singlet and triplet states that lead to quite different isomeric energy splittings. For instance, the proximity of the unpaired electrons in *o*-benzyne allows strong through-space coupling, resulting in a molecule with a fairly closed-shell nature. One may even draw a resonance structure for this isomer containing a triple bond (Figure 6). This interaction significantly stabilizes the singlet while destabilizing the triplet (Δ*E*(ST) (AQCC/TZ) = 38.21 kcal/mol). For comparison, closed shell pentacene has a UED close to 1 and a T1 energy of ∼23 kcal/mol, suggesting that *o*-benzyne may be more closed-shell than pentacene.^136^ For *m*-benzyne, the ground state singlet is quite distorted, likely due to significant through space interaction between the unpaired electrons, leading to the unusual resonance structure shown in Figure 6. Again we see significant stabilization of the singlet (but not as much as for *o*-benzyne; Figure 5) likely due to through-space and through-bond coupling, leading to a (Δ*E*(ST) (AQCC/TZ) = 21.76 kcal/mol).^109^ For *p*-benzyne, through space interaction is unlikely but through bond coupling stabilizes the singlet below the triplet^41,42^ (Δ*E*(ST) (AQCC/TZ) = 5.99 kcal/mol).

### UED

**Figure 8:**
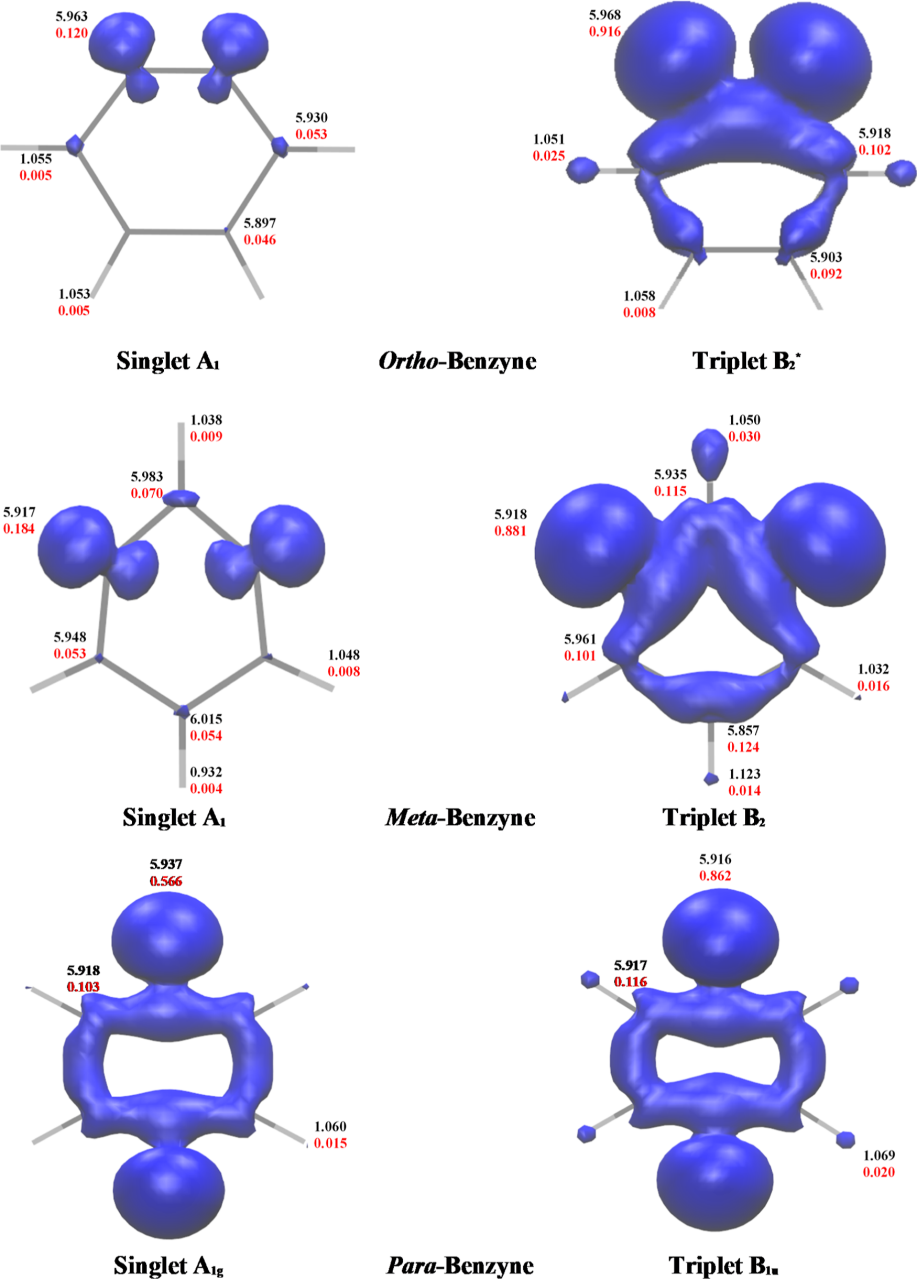
Unpaired electron density plots for the benzynes (Isovalue: 0.006). Mulliken electron population (black) and UED (red) are displayed. MR-AQCC/cc-pVTZ geometries were used for these calculations.

To better gauge the open-shell nature of these isomers, and to compare their electron density distributions, Mulliken electron populations, the number of effectively unpaired electrons (*N*_U_) and the UED were determined using the MR-AQCC/cc-pVTZ wave functions. The UED are visualized in Figure 8. For calibration, for closed shell molecules such as benzene, we expect to see Mulliken values that correlate roughly with the number of electrons “assigned” to particular atoms, i.e. ∼6 and ∼0.8 for C and H, respectively. The UED plots provide a qualitative description of the amount of interaction between the unpaired electrons as well as their delocalization throughout the molecule. *N*_U_ values quantify these effects. The total number of unpaired electrons (*N*_U_) and the unpaired electron density^137^ were determined using eq 1 via the nonlinear formula established by Head-Gordon,^131^ which involves summing over all NO occupations
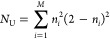
1where *n*_*i*_ is the occupation of the *i*th NO and *M* is the number of NOs. The UED values and plots provide good estimations of the relative difference in unpaired electron density between the singlet and triplet states.

For *o-*benzyne, the total unpaired electron density is 0.457 and 2.285 for the singlet and triplet states, respectively, demonstrating that there is significantly more electron interaction in the singlet state, leading to less UED and consequent stabilization of the singlet state. The unpaired electron density is distributed mostly among the carbon atoms, with very little in the hydrogen atoms in both the singlet and triplet state. In the singlet state for *o-*benzyne, the diradical carbons 1 and 2 (0.120 electrons) have twice the contribution compared with carbons 3 and 6 (0.053) or carbons 4 and 5 (0.046). In the triplet state, however, the diradical atoms (0.916) have approximately ten times as much UED as the other carbon atoms (0.102, 0.092). In contrast to the singlet state, the UED on the diradical carbons for the triplet state is much closer to one, indicative of very little interaction between the radical electrons.

*m-*benzyne displays similar density patterns as *o*-benzyne. For instance, in *m*-benzyne, the radical-containing carbon atoms have 0.184 unpaired electrons in the singlet state as compared to 0.881 in the triplet state. The total UED is 0.626 for the singlet state and 2.280 for the triplet state. The singlet state total UED is slightly for *m-*benzyne as compared to *o-*benzyne, while the triplet state total UED is about the same for both isomers. The higher UED for the singlet state of *m*-benzyne, relative to *o*-benzyne, suggests that there is less radical electron interaction in the singlet state of *m-*benzyne, leading to a smaller adiabatic and vertical gap compared with *o-*benzyne. The plots in Figure 8 show the unpaired electron density in blue, from which we can see that the UED is significantly higher in the triplet state of *m*-benzyne.

*p-*benzyne, in contrast, shows a relative unpaired electron density of 0.566 for the radical carbons in the singlet state and 0.862 for the radical carbons in the triplet state. The difference between the UED of the two states is less than the difference seen in either *o-* or *m-*benzyne. Similarly, the total UED is 1.604 for the singlet state and 2.270 for the triplet state. Although the UED in the triplet remains close to 2 as for the other benzyne isomers, the UED in the singlet state increases significantly. Once again, the location of the radicals affects the extent of their interaction. The fact that *p-*benzyne has a higher UED in the singlet state is a confirmation that the smaller S–T gap, relative to *o-* and *m-*benzyne, is due to the singlet state not being as stabilized by radical interactions.

## Conclusions

We have characterized the geometries, and the adiabatic and vertical S–T gaps, for the lowest two states of *o-*, *m-*, and *p-*benzyne using multireference methods MC-SCF, MR-CISD, MR-CISD+Q and MR-AQCC. An (8,8) CAS was used that included the σ, σ*, π and π* orbitals. Comparison with previously published data shows that these methods are capturing the various open-shelled nature of the different isomers and produce energies that are in good agreement with experiment. The distance between the unpaired electrons governs the extent of radical interaction, and consequent stabilization of the singlet state. Evidence of this can be seen through geometry and UED analysis.

## Supporting Information Available

Additional computational details including a graphical comparison of the experimental heats of formation with the singlet–triplet energies for the benzynes, Cartesian coordinates for all species, adiabatic gaps using the DZ basis set and absolute energies for all geometry optimized species (PDF)
