# Supplementary material for: Multireference Averaged Quadratic Coupled Cluster (MR-AQCC) Study of the Geometries and Energies for ortho-, meta- and para-Benzyne
Source: J Phys Chem A. 2024 Sep 6;128(37):7816–29. doi: 10.1021/acs.jpca.4c04099 (PMC11421082; doi:10.1021/acs.jpca.4c04099)
Supplement: Supplementary file 1 — jp4c04099_si_001.pdf [file jp4c04099_si_001.pdf]

A Multireference Averaged Quadratic Coupled Cluster (MR-AQCC) Study of the Geometries and Energies for *Ortho* -, *Meta* - and *Para*-benzyne

Khanh Vu,[1] Joshua Pandian,[1] Boyi Zhang,[1] Christina Annas,[1] Anna J. Parker,[1] John S. Mancini,[1] Evan B. Wang,[1] Diomedes Saldana-Greco,[1] Emily S. Nelson,[1] Greg Springsted,[1] Hans Lischka,[2] Felix Plasser[3] and Carol A. Parish[1]*

[1]Department of Chemistry, Gottwald Center for the Sciences, University of Richmond, Richmond VA, 23173 US, cparish@richmond.edu

[2]Department of Chemistry and Biochemistry, Texas Tech University, Lubbock, Texas, 79409 US, hans.lischka@ttu.edu

[3]Department of Chemistry, Loughborough University School of Science, Ashby Road, Loughborough, Leicestershire, UK LE11 3TU, f.plasser@lboro.ac.uk

*To whom correspondence should be addressed. E-mail: cparish@richmond.edu, phone (804) 484-1548, fax (804) 287-1897.

Supporting Information:

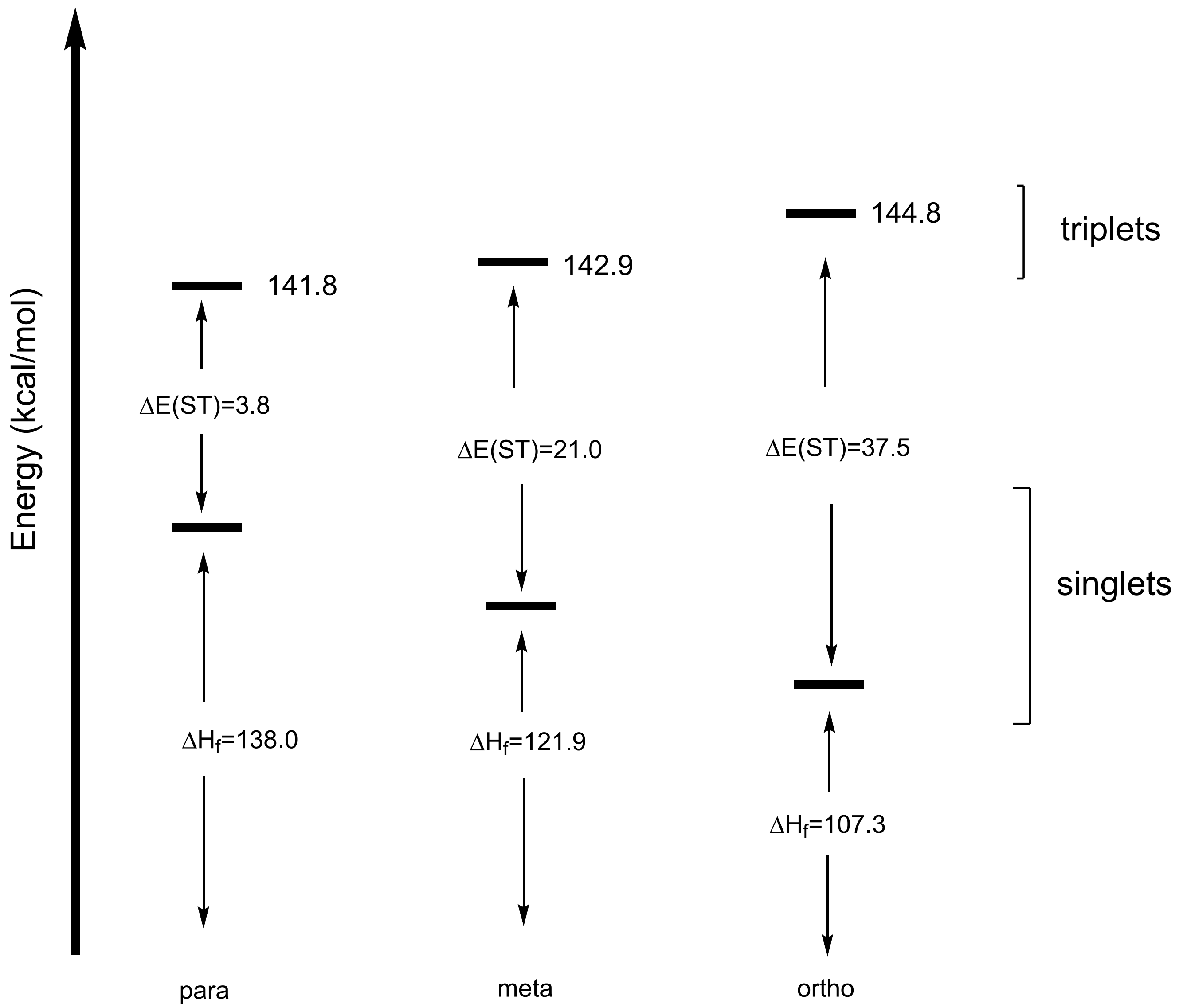

Table S1. A comparison of the experimental heats of formation (ΔH_f) and AQCC/cc-pVTZ singlet-triplet splitting ($\Delta E(ST)$) energies for the benzynes.

Note: vertical excitations were not computed at the DZ level of theory.

I. Adiabatic Gaps using the DZ basis set

Table S1. *Ortho* -benzyne MCSCF, MR-CISD, MR-CISD+Q, and MR-AQCC adiabatic gaps (kcal/mol) from the singlet A_1 ground state using a CAS(8,8) reference wave function and the cc-pVDZ basis set.

State	Dominant Configuration in AQCC	MCSCF E_{exc}	MR-CISD E_{exc}	MR-CISD+Q E_{exc}	AQCC E_{exc}
$^1A_1$	64.7% $\pi_1^2 \pi_2^2 \pi_3^2 \sigma^2$				
	4.6% $\pi_1^2\pi_2^2\pi_3^2(\sigma^*)^2$	33.86	34.97	34.94	34.58
$^3B_2$	68.7% $\pi_1^2\pi_2^2\pi_3^2\sigma^1(\sigma^*)^1$				

All energies are in table S5.

Table S2. *Meta* -benzyne MCSCF, MR-CISD, MR-CISD+Q, and MR-AQCC adiabatic gaps (kcal/mol) from the singlet A_1 ground state using a CAS(8,8) reference wavefunction and the cc-pVDZ basis set.

State	Dominant Configuration in AQCC	MCSCF E_{exc}	MR-CISD E_{exc}	MR-CISD+Q E_{exc}	AQCC E_{exc}
$^1A_1$	61.5% $\pi_1^2\pi_2^2\pi_3^2\sigma^2$				
	8.0% $\pi_1^2\pi_2^2\pi_3^2(\sigma^*)^2$	15.24	18.41	19.81	19.40
$^3B_2$	68.7% $\pi_1^2\pi_2^2\pi_3^2\sigma^1(\sigma^*)^1$				

All energies are in table S5.

Table S3. *Para*-benzyne MCSCF, MR-CISD, MR-CISD+Q, and MR-AQCC adiabatic gaps (kcal/mol) from the singlet A_1 ground state using a CAS(8,8) reference wave function and the cc-pVDZ basis set.

State	Dominant configuration in AQCC	MCSCF E_{exc}	MR-CISD E_{exc}	MR-CISD+Q E_{exc}	AQCC E_{exc}
^1A_g	48.2% $\pi_1^2 \pi_2^2 \pi_3^2 (\sigma^*)^2$				
	20.2%$\pi_1^2\pi_2^2\pi_3^2(\sigma)^2$	2.74	3.86	5.10	5.78
$^3B_{3u}$	69.8% $\pi_1^2 \pi_2^2 \pi_3^2 \sigma^1 (\sigma^*)^1$				

All energies are in table S5.

II. Energies

Table S4. Absolute energies of the calculations using CAS (8,8) and cc-pVTZ basis set.

Isomers	MCSCF	MR-CISD	MR-CISD+Q	AQCC

$^{1}A_{1}$ *Ortho*	-229.59780	-230.30567	-230.45654	-230.44967
$^{3}B_{2}$ *Ortho*	-229.54122	-230.24499	-230.39544	-230.38878
$^{3}B_{2}$ *Ortho* (Vert.)	-229.51617	-230.22107	-230.37122	-230.36392
$^{1}A_{1}$ *Meta*	-229.57036	-230.28228	-230.43669	-230.42953
$^{3}B_{2}$ *Meta*	-229.54526	-230.25051	-230.40175	-230.39485
$^{3}B_{2}$ *Meta* (Vert.)	-229.52050	-230.22907	-230.38105	-230.37372
$^{1}A_{g}$ *Para*	-229.55108	-230.25665	-230.40764	-230.40149
$^{3}B_{3u}$ *Para*	-229.54685	-230.25065	-230.39950	-230.39195
$^{3}B_{3u}$ *Para* (Vert.)	-229.54357	-230.24748	-230.39729	-230.38937

Table S5. Absolute energies of the calculations using CAS (8,8) and cc-pVDZ basis set.

Isomers	MCSCF	MR-CISD	MR-CISD+Q	AQCC
$^{1}A_{1}$ *Ortho*	-229.54225	-230.13122	-230.25563	-230.24754
$^{3}B_{2}$ *Ortho*	-229.48828	-230.07549	-230.19995	-230.19244
$^{1}A_{1}$ *Meta*	-229.51630	-230.10965	-230.23695	-230.22896
$^{3}B_{2}$ *Meta*	-229.49201	-230.08031	-230.20538	-230.19805
$^{1}A_{g}$ *Para*	-229.49792	-230.08660	-230.21150	-230.20436
$^{3}B_{3u}$ *Para*	-229.49355	-230.08045	-230.20337	-230.19515

III. Geometries (cc-pVTZ)

Ortho-benzyne | MR-AQCC | Singlet

C	0.000000	0.631446	-1.232145
C	0.000000	-0.631446	-1.232145

C	0.000000	1.459106	-0.117768
C	0.000000	-1.459106	-0.117768
C	0.000000	0.703258	1.071565
C	0.000000	-0.703258	1.071565
H	0.000000	2.537446	-0.118293
H	0.000000	-2.537446	-0.118293
H	0.000000	1.225266	2.018880
H	0.000000	-1.225266	2.018880

Ortho-benzyne | MR-CISD | Singlet

C	0.000000	0.626238	-1.228690
C	0.000000	-0.626238	-1.228690
C	0.000000	1.448484	-0.113362
C	0.000000	-1.448484	-0.113362
C	0.000000	0.703267	1.066171
C	0.000000	-0.703267	1.066171
H	0.000000	2.519674	-0.117121
H	0.000000	-2.519674	-0.117121
H	0.000000	1.218692	2.008740
H	0.000000	-1.218692	2.008740

Ortho-benzyne | MCSCF | Singlet

C	0.000000	0.625057	-1.239652
C	0.000000	-0.625057	-1.239652
C	0.000000	1.447402	-0.109275
C	0.000000	-1.447402	-0.109275
C	0.000000	0.709536	1.068028
C	0.000000	-0.709536	1.068028
H	0.000000	2.517849	-0.117243
H	0.000000	-2.517849	-0.117243
H	0.000000	1.223119	2.011190
H	0.000000	-1.223119	2.011190

Ortho-benzyne | AQCC | Triplet

C	0.000000	0.699851	-1.297862
C	0.000000	-0.699851	-1.297862
C	0.000000	1.408640	-0.112227
C	0.000000	-1.408640	-0.112227
C	0.000000	0.696967	1.099508
C	0.000000	-0.696967	1.099508

H	0.000000	2.490124	-0.116514
H	0.000000	-2.490124	-0.116514
H	0.000000	1.238321	2.034590
H	0.000000	-1.238321	2.034590

Ortho-benzyne | CISD | Triplet

C	0.000000	0.696092	-1.287764
C	0.000000	-0.696092	-1.287764
C	0.000000	1.405129	-0.109730
C	0.000000	-1.405129	-0.109730
C	0.000000	0.693687	1.093246
C	0.000000	-0.693687	1.093246
H	0.000000	2.478709	-0.115627
H	0.000000	-2.478709	-0.115627
H	0.000000	1.228514	2.024001
H	0.000000	-1.228514	2.024001

Ortho-benzyne | MCSCF | Triplet

C	0.000000	0.698097	-1.291210
C	0.000000	-0.698097	-1.291210
C	0.000000	1.408932	-0.110377
C	0.000000	-1.408932	-0.110377
C	0.000000	0.695144	1.095588
C	0.000000	-0.695144	1.095588
H	0.000000	2.481812	-0.116360
H	0.000000	-2.481812	-0.116360
H	0.000000	1.228784	2.026602
H	0.000000	-1.228784	2.026602

Para-benzyne | MR-AQCC | Singlet

C	0.000000	0.000000	1.354621
C	0.000000	0.000000	-1.354621
C	0.000000	1.211368	0.716086
C	0.000000	-1.211368	0.716086
C	0.000000	1.211368	-0.716086
C	0.000000	-1.211368	-0.716086
H	0.000000	2.161367	1.228456
H	0.000000	-2.161367	1.228456
H	0.000000	2.161367	-1.228456
H	0.000000	-2.161367	-1.228456

Meta-benzyne | AQCC | Singlet

C	0.000000	1.176962	-0.658486
C	0.000000	-1.176962	-0.658486
C	0.000000	1.044044	0.711348
C	0.000000	-1.044044	0.711348
C	0.000000	0.000000	1.603770
C	0.000000	0.000000	-1.421004
H	0.000000	2.154840	-1.116736
H	0.000000	-2.154840	-1.116736
H	0.000000	-0.000000	2.678648
H	0.000000	-0.000000	-2.504523

Meta-benzyne | MR-CISD | Singlet

C	0.000000	1.173145	-0.656956
C	0.000000	-1.173145	-0.656956
C	0.000000	1.048600	0.709263
C	0.000000	-1.048600	0.709263
C	0.000000	-0.000000	1.585248
C	0.000000	0.000000	-1.407765
H	0.000000	2.143939	-1.112872
H	0.000000	-2.143939	-1.112872
H	0.000000	-0.000000	2.652757
H	0.000000	-0.000000	-2.483462

Meta-benzyne | MCSCF | Singlet

C	0.000000	1.182911	-0.654090
C	0.000000	-1.182911	-0.654090
C	0.000000	1.086875	0.720261
C	0.000000	-1.086875	0.720261
C	0.000000	0.000000	1.561318
C	0.000000	0.000000	-1.392674
H	0.000000	2.145828	-1.125793
H	0.000000	-2.145828	-1.125793
H	0.000000	0.000000	2.627916
H	0.000000	0.000000	-2.467401

Meta-benzyne | MR-AQCC | Triplet

C	0.000000	1.224730	-0.641356
C	0.000000	-1.224730	-0.641356
C	0.000000	1.170266	0.739917
C	0.000000	-1.170266	0.739917
C	0.000000	-0.000000	1.482164
C	0.000000	0.000000	-1.328170
H	0.000000	2.159414	-1.180895
H	0.000000	-2.159414	-1.180895
H	0.000000	0.000000	2.563157
H	0.000000	-0.000000	-2.409823

Meta-benzyne | MR-CISD | Triplet

C	0.000000	1.215362	-0.640093
C	0.000000	-1.215362	-0.640093
C	0.000000	1.163632	0.735539
C	0.000000	-1.163632	0.735539
C	0.000000	-0.000000	1.475498
C	0.000000	0.000000	-1.326475
H	0.000000	2.146787	-1.171467
H	0.000000	-2.146787	-1.171467
H	0.000000	0.000000	2.548638
H	0.000000	-0.000000	-2.400478

Meta-benzyne | MCSCF | Triplet

C	0.000000	1.215352	-0.641294
C	0.000000	-1.215352	-0.641294
C	0.000000	1.169682	0.740249
C	0.000000	-1.169682	0.740249
C	0.000000	-0.000000	1.478574
C	0.000000	0.000000	-1.331890
H	0.000000	2.148024	-1.169911
H	0.000000	-2.148024	-1.169911
H	0.000000	0.000000	2.550927
H	0.000000	-0.000000	-2.405305

Para-benzyne | CISD | Singlet

C	0.000000	-0.000000	1.345198
C	0.000000	0.000000	-1.345198

C	0.000000	1.209489	0.707253
C	0.000000	-1.209489	0.707253
C	0.000000	1.209489	-0.707253
C	0.000000	-1.209489	-0.707253
H	0.000000	2.145278	1.231290
H	0.000000	-2.145278	1.231290
H	0.000000	2.145278	-1.231290
H	0.000000	-2.145278	-1.231290

Para-benzyne | MCSCF | Singlet

C	0.000000	-0.000000	1.348269
C	0.000000	-0.000000	-1.348269
C	0.000000	1.217099	0.704017
C	0.000000	-1.217099	0.704017
C	0.000000	1.217099	-0.704017
C	0.000000	-1.217099	-0.704017
H	0.000000	2.144839	1.241711
H	0.000000	-2.144839	1.241711
H	0.000000	2.144839	-1.241711
H	0.000000	-2.144839	-1.241711

Para-benzyne | AQCC | Triplet

C	0.000000	-0.000000	1.325500
C	0.000000	0.000000	-1.325500
C	0.000000	1.234119	0.703325
C	0.000000	-1.234119	0.703325
C	0.000000	1.234119	-0.703325
C	0.000000	-1.234119	-0.703325
H	0.000000	2.158632	1.262130
H	0.000000	-2.158632	1.262130
H	0.000000	2.158632	-1.262130
H	0.000000	-2.158632	-1.262130

Para-benzyne | CISD | Triplet

C	0.000000	0.000000	1.321929
C	0.000000	-0.000000	-1.321929
C	0.000000	1.227416	0.698952
C	0.000000	-1.227416	0.698952
C	0.000000	1.227416	-0.698952
C	0.000000	-1.227416	-0.698952

H	0.000000	2.144550	1.256066
H	0.000000	-2.144550	1.256066
H	0.000000	2.144550	-1.256066
H	0.000000	-2.144550	-1.256066

Para-benzyne | MCSCF | Triplet

C	0.000000	-0.000000	1.331948
C	0.000000	0.000000	-1.331948
C	0.000000	1.228565	0.699606
C	0.000000	-1.228565	0.699606
C	0.000000	1.228565	-0.699606
C	0.000000	-1.228565	-0.699606
H	0.000000	2.145113	1.256614
H	0.000000	-2.145113	1.256614
H	0.000000	2.145113	-1.256614
H	0.000000	-2.145113	-1.256614
